# Ex vivo cardiac DTI: on the effects of diffusion time and b-value

**DOI:** 10.1186/1532-429X-16-S1-P77

**Published:** 2014-01-16

**Authors:** Martijn Froeling, Valentina Mazzoli, Aart J Nederveen, Peter R Luijten, Gustav J Strijkers

**Affiliations:** 1 Department of Radiology, University Medical Center, Utrecht, Netherlands; 2 Department of Biomedical Engineering, Biomedical NMR, Eindhoven University of Technology, Eindhoven, Netherlands; 3 Department of Radiology, Academic Medical Center, Amsterdam, Netherlands

## Background

For in-vivo an ex-vivo studies of myocardial structure, generally a spin echo (SE) or stimulated echo (STE) based sequence is used. Diffusion weighting is reported with a large range of diffusion times (50 and 1000 ms). Furthermore, in-vivo studies generally use low b-values (300 s/mm^2^) whereas ex-vivo experiments allow for much higher b-values. For interpretation of in-vivo cardiac DTI a understanding of the effects of sequence parameters on the derived diffusion parameters is needed. In this study, we therefore studied the impact of diffusion time and b-value on these parameters.

## Methods

Experiments were performed on ex-vivo pig hearts stored in 4% paraformaldehyde for at least three weeks. 24 hours before scanning, the hearts were rinsed and placed in a water bath for rehydration. Data was acquired with a 3T scanner (Philips, Achieva) using an 8-channel head coil with a DW-STE sequence with multi-shot EPI readout. Acquisition parameters were: FOV: 128 × 128 mm^2^, voxel size: 2 × 2 × 2 mm^3^, TE/TR: 55/3500 ms, 9 slices (5 mm gap), 14 b-values (250 to 3500 s/mm^2^), TM: 30, 50, 70, 110, 150, 200, 250 and 300 ms, 8 gradient directions. The data was registered to the average b = 0 images followed by Rician noise suppression. The data was fitted using a single tensor (LLS) and a two-tensor model (NLS). The latter was constrained to follow the fiber directions found in the single tensor model.

## Results

**Figure 1:**
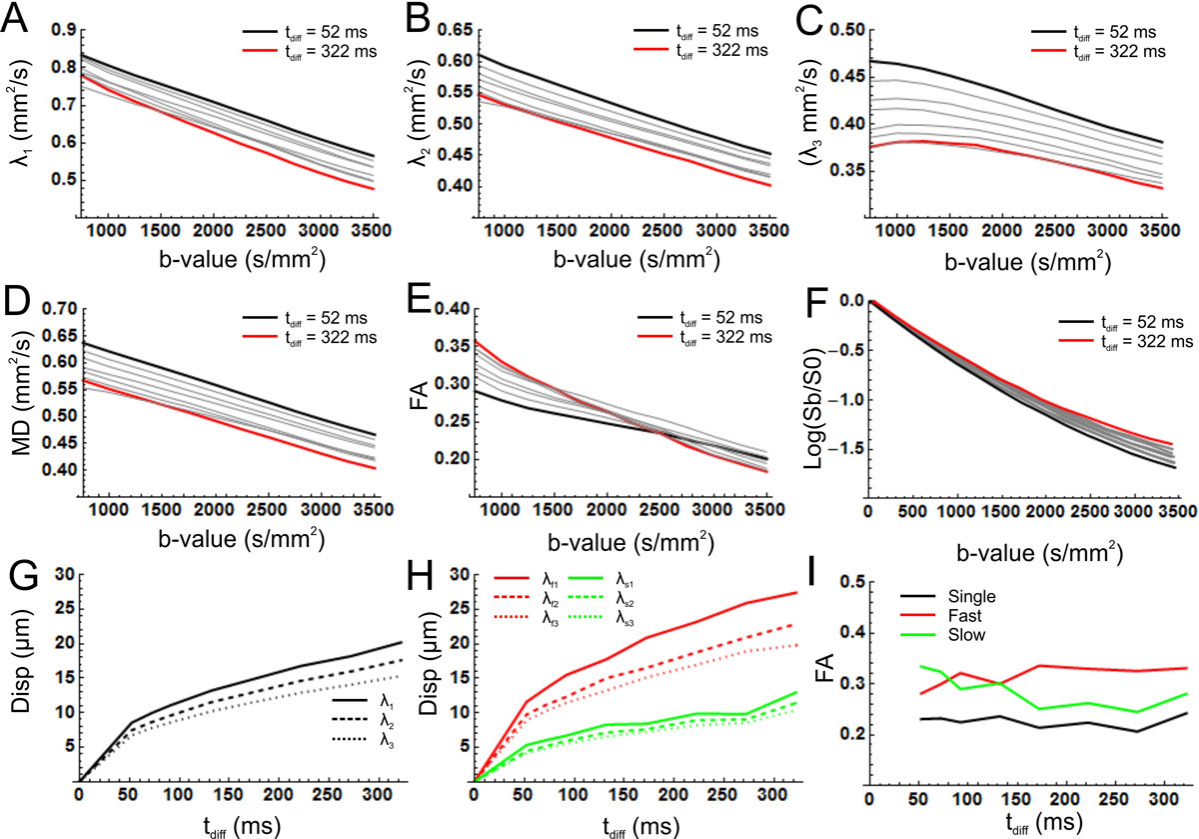
**A-E) Diffusion parameters as function of b-value for different mixing times (red: T_diff _= 52 ms, black: T_diff _= 322 ms)**. F) Normalized signal as a function of b-value. G-I) Mean displacement as a function of the diffusion time for the three eigenvalues (G single tensor fit. H two tensor fit). I) FA as a function of the diffusion time for the one and two tensor fits.

**Figure 2:**
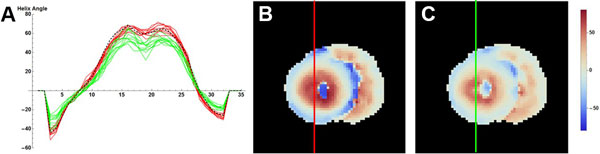
**A) Helix angel for a cross sectional line as indicated in figures B and C (Black: based on all b-values and diffusion times**. Red: all b-values for a diffusion time of 52 ms. Green: all b-values for a diffusion time of 322 ms). B) helix-angle map for T_diff _= 52 ms. C) helix-angle map for T_diff _= 322 ms.

There was a non-linear dependency of the signal on the applied b-value for all diffusion times (Figure 1E), which was more apparent for longer diffusion times. As a result the DTI parameters also depended on b-value and diffusion time (Figure 1A to 1E). Furthermore the helix angle decreased for longer diffusion times (Figure 2). The mean displacement as a function of the diffusion time showed that the diffusion is restricted (Figure 1G). The two tensor model showed that there are two compartments, a slow and a fast (Figure 1H), where the fast components has a slightly higher FA (Figure 1I).

## Conclusions

We have shown that de DTI derived parameters and fiber tractography depend on both diffusion time and b-value. Furthermore the two-tensor fit shows two compartments with different diffusion properties. Here the compartment with the slower diffusion could be within the muscle fibers where the actin and myosin filaments are tightly packed whereas the faster diffusion compartment could be the space surrounding the sarcomeres. It is important to consider these findings in the interpretation of in-vivo cardiac DTI data.

## Funding

Not applicable.

